# Mendelian randomization shows depression increases the risk of type 2 diabetes

**DOI:** 10.3389/fgene.2023.1181851

**Published:** 2023-08-24

**Authors:** Heejin Jin, Sanghun Lee, Sungho Won

**Affiliations:** ^1^ Institute of Health and Environment, Seoul National University, Seoul, Republic of Korea; ^2^ Department of Medical Consilience, Graduate School, Dankook University, Yongin-si, Republic of Korea; ^3^ Department of Public Health Science, Seoul National University and Institute of Health and Environment, Seoul National University, Seoul, Republic of Korea

**Keywords:** depression, type 2 diabetes mellitus, bipolar disorder, schizophrenia, mendelian randomization analysis, causality

## Abstract

**Introduction:** Type 2 diabetes (T2D) is associated with severe mental illnesses (SMIs), such as schizophrenia, bipolar disorder, and depression. However, causal relationships between SMIs and T2D remain unclear owing to potential bias in observational studies. We aimed to characterize the causal effect of SMI liability on T2D using two-sample Mendelian randomization (MR).

**Methods:** The causality between liability to SMI and T2D was investigated using the inverse-variance weighted (IVW), MREgger, MR-Egger with a simulation extrapolation, weighted median, and the MR pleiotropy residual sum and outlier method. Similarly, we performed additional MR which can detect the reverse causation effect by switching exposure and outcome for T2D liability for SMI. To further consider pleiotropic effects between SMIs, multivariable MR analysis was performed after accounting for the other traits.

**Results:** In the univariable IVW method, depression showed a causal effect on T2D (odds ratio [OR]: 1.128, 95% confidence interval [CI]: 1.024–1.245, *p* = 0.014). Multinomial MR more strongly supported these results (IVW OR: 1.197, 95% CI: 1.069, 1.340, *p* = 0.002; MR-Egger OR: 1.198, 95% CI: 1.062, 1.349, *p* = 0.003). Bidirectional MR showed absence of reversecausality between depression and T2D. However, causal relationship of bipolar and schizophrenia on T2D was not detected.

**Discussion:** Careful attention is needed for patients with depression regarding T2D prevention and treatment.

## Background

To date, several epidemiologic studies have suggested a link between type 2 diabetes (T2D) and severe mental illnesses (SMIs), including bipolar disorder (BPD), schizophrenia (SCZ), and depression (Vancampfort et al., 2016). The prevalence of T2D among individuals with BPD and SCZ has been estimated to be 8%–17% and 16%–25%, respectively; furthermore, some studies have reported that adults with depression have a 37% increased risk of developing T2D (Dixon et al., 2000; Anderson et al., 2001; Regenold et al., 2002). The side effects of the medications used for treating SMI, unhealthy lifestyle behaviors of patients with SMI, and hypothalamic–pituitary–adrenal axis dysregulation could contribute to the association of SMI with T2D (Scott and Happell, 2011; Enger et al., 2013; Vancampfort et al., 2015).

In contrast, T2D also affects mental health. Some studies have reported that the risk of depression increases in people with T2D (Nouwen et al., 2010), while the prevalence of SCZ is higher in patients with T2D than in the general population (Huang et al., 2018). Furthermore, T2D and prediabetes may be risk factors in patients with BPD (Hajek et al., 2015), and diabetes affects mental health (Feng and Astell-Burt, 2017). Furthermore, it is also well known that many diseases have downstream effects on mental health as part of a prodromal phase. However, owing to potential biases, such as confounding and reverse causation in these studies, the true causality between liability to SMI and T2D remains unclear. Moreover, the difference between the age at onset of T2D and SMI makes it extremely challenging to infer a causal relationship between them. In general, heritability for T2D is typically between 25% and 80%, with onset at a younger age indicating a stronger genetic component, while SMI usually occurs in young adulthood (20s–30s) with high heritability (80%–85% for BPD, 80% for SCZ, and 31%–42% for depression) (Sullivan et al., 2000; Purcell et al., 2009). Accordingly, both directions warrant investigation. Two-sample Mendelian randomization (MR) studies, widely adopted using genetic variants, such as single nucleotide polymorphisms (SNPs) for instrumental variable IV) analysis, should pinpoint the causal relationship between liability to SMI and T2D (Burgess et al., 2016a). Anti-psychiatric treatments may cause bias, and some SNPs that are used as potential IVs may be strongly associated with side effects; if this is the case, genetically determined depression and anti-depressive efficacy may be responsible for the estimated causal effect of SMIs on T2D (Zhang et al., 2021a; Zhang et al., 2021b; Zhang and Baranova, 2021). Therefore, it is crucial to explicitly clarify the roles of shared genes and pleiotropy in this study, which seeks to elucidate the causal relationship between mental illnesses and T2D. For these reasons, in our MR analysis, the pleiotropic effects were considered from multiple aspects, and various sensitivity MR analyses were applied (Hou et al., 2020; Jin et al., 2020; Wang et al., 2021). Consequently, we investigated the causality between SMI (BPD, SCZ, and depression) liability and T2D using genome-wide summary statistics in a meta-analysis of a large population of European individuals through a two-sample bidirectional MR study.

## Methods

**FIGURE 1:**
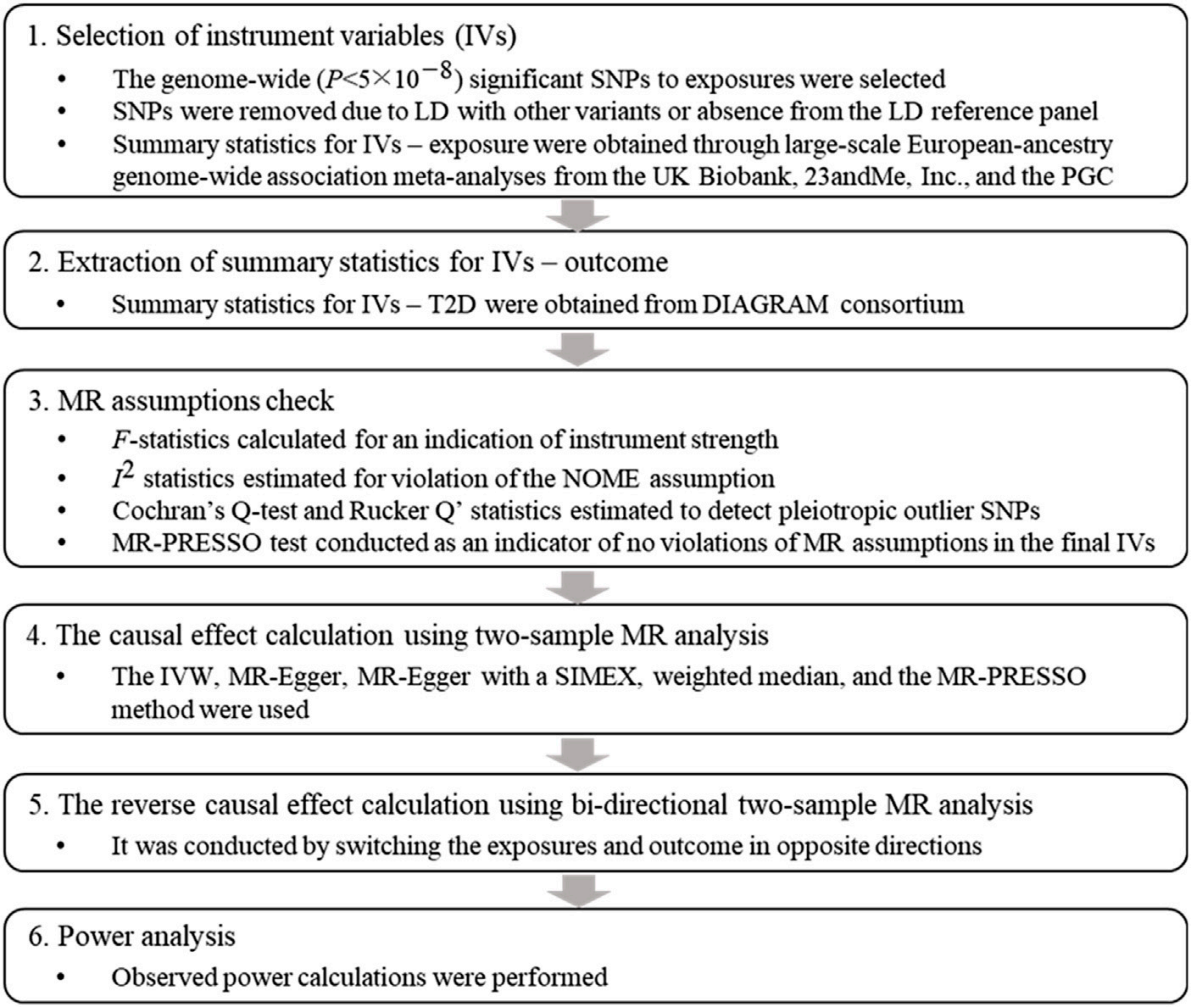
Flowchart of the Mendelian randomization study. Abbreviations: SNP: single nucleotide polymorphism, LD: linkage disequilibrium, PGC: psychiatric genomics consortium, T2D: type 2 diabetes, DIAGRAM: DIAbetes Genetics Replication And Meta-analysis, MR: Mendelian randomization, MR-PRESSO: Mendelian Randomization Pleiotropy RESidual Sum and Outlier, IVW: inverse variance weighted, SIMEX: simulation extrapolation.

Two-sample MR analysis was conducted, and Figure 1 displays a flowchart describing the overall procedure.

### Exposure datasets: summary statistics of genetic association analyses for BPD, depression, and SCZ

The first exposure dataset is the summary statistics of the genetic association analyses for BPD with 20,352 cases and 31,358 controls (Stahl et al., 2019). The characteristics of the data is shown in Table 1. Cases were required to meet international consensus criteria (Diagnostic and Statistical Manual of Mental Disorders [DSM-IV] and International Classification of Diseases, Ninth or 10th Revision [ICD-9 or ICD-10]) for a BPD lifetime diagnosis. To select an appropriate instrument, “ld_clump” function of R package “ieugwasr” was used. Among the genome-wide (*p*-value *p*) < 5 $\times 10^{-8}$ ) significant SNPs, some variants were removed due to linkage disequilibrium (LD) with other variants or absence from the LD reference panel. Thus, 16 genome-wide SNPs associated with BPD liability were identified after LD pruning (distance <10,000 kb or LD $r^{2}$ <0.001). The above series of processes will be referred to as “IV QC (IV quality control)" in the remainder of this article. After the removal of SNPs nominally associated with T2D (*p* < 0.05), 11 BPD-associated SNPs were available. The second exposure dataset is the summary statistics of the genetic association analyses for depression with 246,363 cases and 561,190 controls (Table 1) (Howard et al., 2019). The definition of depression was different in each cohort. In the UK Biobank, the following three depression phenotypes were used: 1) self-reported help-seeking for problems with nerves, anxiety, tension, or depression, 2) self-reported depressive symptoms, and 3) depression identified from hospital admission records. In the case of 23andMe, Inc., a self-reported clinical diagnosis of depression was used. Finally, cases were required to meet various international consensus criteria (DSM-IV, ICD-9, or ICD-10) in the Psychiatric Genomics Consortium. A total of 50 were selected as IV candidates after IV QC. We checked the pleiotropic effect of those SNPs, and eight SNPs associated with T2D (*p* < 0.05) were eliminated. Thus, 42 SNPs were associated with depression liability. The third exposure dataset is the summary statistics of the genetic association analyses for SCZ with 33,640 cases and 43,456 controls (Table 1) (Lam et al., 2019). From this genome-wide association study (GWAS), we exclusively considered the European population analysis to satisfy the two-sample MR assumption of having the same underlying population in both exposure and outcome GWAS. Cases with clinical diagnoses (not self-reported) of SCZ or schizoaffective disorder were included in our study. A total of 83 SNPs was associated with SCZ after IV QC. Eleven SNPs were associated with T2D (*p* < 0.05), and the remaining 72 SCZ liability-associated SNPs were used for our MR analyses.

**TABLE 1:** Description of GWAS data for MR study.

Variable	PMID	Consortium	Sample size (case/control)	Population	SNPs
BPD	31,043,756	NA	20,352/31,358	European, North america, Australia	9,372,253
Depression	30,718,901	UK Biobank, 23andMe, Inc, PGC	246,363/561,190	European	8,098,588
SCZ	31,740,837	PGC	33,640/43,456	European	13,942,226
T2D	28,566,273	DIAGRAM	26,676/132,532	European	12,100,000

Abbreviations: BPD: bipolar disorder; SCZ: schizophrenia; T2D: type 2 diabetes, PGC: psychiatric genomics consortium; DIAGRAM: DIAbetes Genetics Replication And Meta-analysis Consortium; NA: not available.

### Outcome datasets: summary statistics of genetic association analyses for T2D

To avoid inflated type-1 error rates and false-positive findings due to sample overlap in two-sample MR (Jin et al., 2020), we obtained summary statistics for the association of SNP with T2D from the DIAbetes Genetics Replication And Meta-analysis Consortium stage 1 meta-analyses with 26,676 cases and 132,532 controls (Table 1) (Scott et al., 2017). T2D diagnosis was based on diagnostic fasting glucose (≥7 mmol/L) or hemoglobin A1c levels (≥6.5%), hospital discharge diagnosis, use of oral anti-diabetic medication, or self-reports. Summary statistics of genome-wide association analyses for 12.1 million SNPs were available and were considered for our MR analyses. When T2D status is considered as an exposure in reverse MR analysis, a total of 35, 39 and 43 SNPs were finally used as instrument variables for BPD, depression and SCZ, respectively.

### Genetic correlation

The genetic correlation (r_g_) between target traits was estimated by bivariate linkage disequilibrium score regression (LDSC) (Yengo et al., 2018). The LD reference panel for the analysis was obtained from the 1000 Genomes Project based on European ancestry information.

### MR analysis

Two-sample MR requires three strong basic assumptions as follows: (ⅰ) a strong association between IVs and intermediate exposure, (ⅱ) IVs independent of confounders, and iii) IVs that do not directly affect the outcome. Additionally, sensitivity MR requires the “NO Measurement Error” (NOME) assumption and InSIDE assumption (INstrument Strength Independent of Direct Effect) (Jin et al., 2020). *F*-statistics provided an indication of instrument strength, and *F* > 10 indicated that the analysis was unlikely to suffer from weak instrument bias (Burgess et al., 2011). A degree of violation of the NOME assumption was quantified using $I^{2}$ statistics, while $I^{2}$ >90 indicated lower estimate dilution in MR analysis (Bowden et al., 2016). To detect pleiotropic outlier SNPs, we used Cochran’s Q-test in the inverse-variance weighted (IVW) method and Rucker Q′ statistics in the MR-Egger (Bowden et al., 2018). We further conducted an MR- Pleiotropy RESidual Sum and Outlier (PRESSO) test as an indicator of no violations of MR assumptions in the final instrumental variable sets (Verbanck et al., 2018). Given that no weak instrument bias (*F* > 10) was observed and the three tests (Cochran’s Q-test, Rucker Q′ test, and MR-PRESSO test) indicated no directional pleiotropic bias, the IVW method, which is robust when all SNPs are valid instruments, was applied (Jin et al., 2020). If pleiotropy and outlier SNPs were detected from MR-PRESSO, since IVW is not recommended, several sensitivity analyses were considered to minimize bias (Verbanck et al., 2018). It shows the results of removing outliers in case of the presence of the horizontal pleiotropy effect. The other sensitivity method is the “weighted median method,” which provides valid causal estimates unless >50% of the instruments are invalid (Jin et al., 2020). The median is not affected by outliers; therefore, the weighted median estimate is not sensitive to a pleiotropic genetic variant. Causal effects obtained from the weighted median of the ratio estimates in genetic instruments indicate which smaller standard error receives more weight. The sensitivity MR method used for estimating the causal effect considering the pleiotropic effect is called the MR-Egger method. This method can estimate appropriate causal effects in the presence of pleiotropy effects even if all SNPs are invalid (Jin et al., 2020). When Cochran’s Q-test is rejected or both Cochran’s Q and Rucker’s Q′ tests are rejected, the MR-Egger method is recommended (Jin et al., 2020). If the InSIDE assumption holds, then the slope of the MR-Egger regression provides a causal effect. However, when the $I^{2}$ statistic quantifying the strength of NOME violation for IVs is low ( $I^{2}$ <90) for the MR-Egger method, regression dilution will occur. In cases wherein the NOME assumption was violated, the simulation extrapolation (SIMEX) method was applied to correct attenuation bias (Bowden et al., 2016). However, there is no uniformly powerful and robust MR methods, and each method have advantages for different circumstance. Therefore, according to the IV assumptions, the most recommended method is indicated in bold in Tables 2 and 3.

**TABLE 2:** Genetic correlations between target traits estimated by bivariate LDSC.

Traits	Genetic correlation		
	Intercept±SE	r_g_ ±SE	*p*
**BPD, T2D**	0.025 ± 0.011	−0.001 ± 0.043	0.852
**Depression, T2D**	0.033 ± 0.015	0.143 ± 0.050	4.30 $\times 10^{-3}$
**SCZ, T2D**	0.062 ± 0.011	−0.165 ± 0.034	$1.56\times 10^{-6}$
**BPD, depression**	0.056 ± 0.008	0.353 ± 0.034	$6.54\times 10^{-25}$
**BPD, SCZ**	0.217 ± 0.007	0.741 ± 0.021	$3.13\times 10^{-281}$
**SCZ, depression**	0.043 ± 0.009	0.335 ± 0.031	$1.96\times 10^{-26}$

Abbreviations: LDSC: linkage disequilibrium score regression; BPD: bipolar disorder; SCZ: schizophrenia; T2D: type 2 diabetes; r_g_: genetic correlation; *p*: *p*-value for GC, from LDSC.

The most recommended method is indicated in bold.

**TABLE 3:** Assumption test for instrumental variable sets.

No.	Exposure	Outcome	N	F-Stat	$I^{2}$ (%)	Q-Test	Q’-Test	MR-PRESSO Global Test
1	BPD	T2D	11	32.9	96.9	0.718	0.821	0.695
2	Depression		42	37.9	97.3	0.808	0.788	0.733
3	SCZ		72	41.5	97.6	0.319	0.290	0.704
4	T2D	BPD	35	35.8	97.3	0.684	0.639	0.598
5		depression	39	36.0	97.3	0.714	0.683	0.711
6		SCZ	43	35.8	97.2	<0.001	<0.001	<0.001

Abbreviations: BPD: bipolar disorder; F-stat: F statistics; MR-PRESSO: Mendelian Randomization Pleiotropy RESidual Sum and Outlier; MR-PRESSO, global test: *p*-value for Mendelian Randomization Pleiotropy RESidual Sum and Outlier global test; N: number of instruments; Q-test: *p*-value for the Q-test from inverse-variance weighted; Q′-test: *p*-value for the Q′-test from Mendelian Randomization-Egger; SCZ: schizophrenia; T2D: Type 2 diabetes.

To facilitate the understanding of the methods and analytical procedures, additional figures (scatter, funnel, forest, and leave-one-out sensitivity analysis plot) are shown. The associations of the variants with exposures and outcomes are shown in a scatter plot with several MR-fitted lines. The generated funnel plot shows symmetry, indicating heterogeneity due to horizontal pleiotropy. In addition, the forest plot shows individual estimate between IVs and the risk for T2D, and leave-one-out variant analysis is illustrated in the form of a forest plot. The MR results were rescaled to represent the odds of T2D per doubling of genetic liability to each exposure through multiplying the log causal estimate and 95% confidence interval (CI) by 0.693 and then exponentiating (Verbanck et al., 2018). A statistical significance threshold of Bonferroni-corrected *p* = 1.67 $\times 10^{-2}$ (0.05/3) was used considering bi-directional MR. All statistical analysis was performed using the R package “TwoSampleMR”.

### Statistical power analysis

Observed power calculations were performed using an online tool (https://sb452.shinyapps.io/power/) (Burgess et al., 2020). Proportions of variance in the exposure explained by genetic variants ( $r^{2}$ ) were required for MR power analysis, and 0.08 (BPD), 0.03 (depression), 0.03 (SCZ), and 0.06 (T2D) were used for $r^{2}$ (Scott et al., 2017; Howard et al., 2019; Lam et al., 2019; Stahl et al., 2019).

### Reverse MR analysis

We conducted a bidirectional MR to investigate the presence of reverse-causality between liability to T2D and SMI risk. Among GWAS for 12.1 million SNPs for T2D liability, a total of 35, 43, and 39 SNPs genome-wide, which were significantly associated with T2D liability, had summary GWAS results for BPD, SCZ, and depression, respectively, after LD pruning. The analysis process described in Figure 1, which involved examining the assumption of the instruments and calculating power, was similarly applied to the bidirectional MR analysis.

### Multivariable MR analysis

There is substantial evidence for partial overlap of genetic influences on SMIs (Cardno and Owen, 2014; Schulze et al., 2014). Therefore, the multivariable MR method would be useful to disentangle effects in situations where the three traits are highly related (Burgess and Thompson, 2015). A total of 143 SNPs associated with at least one exposure was used for multivariable MR. To assess instrument strength and heterogeneity, the conditional F-statistic and Cochrane’s Q statistic were calculated (Sanderson et al., 2019). All analysis was performed using the R package “MVMR” and “MendelianRandomization”. If Cochran’s Q test *p* less than 0.05, we used the random-effects multivariable IVW method. In addition, to consider potential pleiotropy, an extension of the MR-Egger method (i.e., multivariable MR-Egger) were applied to identify the causal effect of BPD, depression, SCZ on T2D (Sanderson et al., 2019). Multivariable MR-Egger intercept indicate pleiotropy (intercept *p* < 0.05) and it can also provide a corrected estimate when such pleiotropy exists.

## Results

### Genetic correlation of BPD, depression, and SCZ with T2D

As shown in Table 2, we identified shared genetics between depression and SCZ with T2D. For depression with T2D, the r_g_ was found to be 0.143 with a *p* of 4.30 $\times 10^{-3}$ and for schizophrenia with T2D, the r_g_ was − 0.165 with a *p* of 1.56 $\times 10^{-6}$ . However, there was no genetic association between BPD and T2D (r_g_ = − 0.001, and *p* = 0.852). We also found strong genetic correlation between SMIs (for BPD with depression: r_g_ = 0.353, and *p* = 6.54 $\times 10^{-25}$ ; for BPD with SCZ: r_g_ = 0.741, and *p* = 3.13 $\times 10^{-281}$ ; and for depression with SCZ: r_g_ = 0.335, and *p* = 1.96 $\times 10^{-26}$ ). The colocalization of association signals was visualized through a stacked Manhattan plots in Supplementary Figure S7.

### Effect of liability to BPD on T2D risk

Eleven SNPs associated with BPD, rather than T2D, were used as IVs. All SNP to the exposure and SNP-outcome effects are presented in Supplementary Table S2. We found no evidence of weak instrument bias (*F*-statistic = 32.9), heterogeneity, or outlier pleiotropy (Q-test, *p* = 0.718; Q′-test, *p* = 0.821; MR-PRESSO global test, *p* = 0.695) (Table 3). Additionally, the MR-Egger test indicated no directional pleiotropic bias (intercept *p =* 0.165) or violation of the NOME assumption ( $I^{2}$ = 96.9%) (Table 3). Since all IV assumptions were satisfied, the inverse-variance weighted (IVW) method was considered the most appropriate method to provide unbiased estimates (Bowden et al., 2018; Jin et al., 2020). There was no evidence of a causal effect of BPD liability on T2D (odds ratio [OR]: 1.004, 95% CI: 0.942, 1.070, *p =* 0.892). All other sensitivity analysis also has the OR with CIs crossing 1 (Table 4). In the MR for the reverse causation of T2D liability on BPD, there was no weak instrument bias (*F*-statistic = 35.8) or violation of the NOME assumption ( $I^{2}=$ 97.3%). No evidence of heterogeneity was found in the Q-test (*p =* 0.684), Q′-test (*p* = 0.639), and MR-PRESSO global test (*p =* 0.598) (Table 3). The most appropriate method, IVW, showed no reverse-causal effect (OR: 1.021, 95% CI: 0.979, 1.064, *p =* 0.313) and all other sensitivity analysis showed similar results (Table 5). Given the sample size and $r^{2}$ , a power of 98% and 50.8% was estimated to detect a true OR of 1.100 for BPD on T2D MR and bidirectional MR, respectively. Visualizations for bi-directional MR analysis (scatter, funnel, forest, and leave-one-out sensitivity analysis plots) are shown in Supplementary Figures S1 and S2. In the multivariable MR controlling for depression and SCZ, weak instrument bias is not expected (conditional *F*-statistic = 30.56), and heterogeneity was detected (Cochran’s Q test *p* < 0.05). Random-effects multivariable IVW and multivariable MR-Egger results showed that the effect of BPD liability did not have a causal effect on T2D (Table 6).

**TABLE 4:** Univariable MR results for SMI liability on T2D.

MR Methods	Parameter	N	OR	95% CI	*p*
1. Effect of BPD liability on T2D					
**IVW**	**Estimate**	**11**	**1.004**	**0.942, 1.070**	**0.892**
MR-Egger	Intercept		0.038	−0.015, 0.090	0.165
	Slope		0.990	0.519, 1.128	0.178
MR-Egger (SIMEX)	Intercept		0.003	−0.544, 0.690	0.540
	Slope		0.996	0.935, 1.060	0.917
Weighted median	Estimate		0.987	0.906, 1.075	0.770
MR-PRESSO	Estimate			No outlier	-
2. Effect of depression liability on T2D					
**IVW**	**Estimate**	**42**	**1.128**	**1.024, 1.245**	**0.014**
MR-Egger	Intercept		0.007	−0.018, 0.032	0.566
	Slope		0.953	0.531, 1.711	0.875
MR-Egger (SIMEX)	Intercept		0.004	0.000, 0.008	0.036
	Slope		1.109	1.016, 1.210	0.025
Weighted median	Estimate		1.132	0.988, 1.295	0.072
MR-PRESSO	Estimate			No outlier	-
3. Effect of SCZ liability on T2D					
**IVW**	**Estimate**	**72**	**1.011**	**0.981, 1.040**	**0.463**
MR-Egger	Intercept		0.001	−0.013, 0.013	0.969
	Slope		1.008	0.894, 1.137	0.888
MR-Egger (SIMEX)	Intercept		0.001	−0.002, 0.004	0.427
	Slope		1.010	0.986, 1.041	0.511
Weighted median	Estimate		1.011	0.968, 1.055	0.615
MR-PRESSO	Estimate			No outlier	-

Abbreviations: BPD: bipolar disorder; CI: confidence interval; IVW: inverse-variance weighted; MR: Mendelian randomization; MR-PRESSO: Mendelian Randomization-Pleiotropy RESidual Sum and Outlier; N: number of instruments; OR: odds of the outcome per doubling in the odds of exposure in the population (intercept of MR-Egger and MR-Egger (SIMEX) is on log‐odds scale); SCZ: schizophrenia; SIMEX: simulation extrapolation; T2D: type 2 diabetes.

The most recommended method is indicated in bold.

**TABLE 5:** Univariable reverse MR results for T2D liability on SMI.

MR Methods	Parameter	N	OR	95% CI	*p*
1. Effect of T2D liability on BPD					
**IVW**	**Estimate**	**35**	**1.021**	**0.979, 1.064**	**0.313**
MR-Egger	Intercept		−0.002	−0.026, 0.022	0.890
	Slope		1.035	0.851, 1.258	0.727
MR-Egger (SIMEX)	Intercept		−0.003	−0.011, 0.004	0.428
	Slope		0.995	0.936, 1.056	0.867
Weighted median	Estimate		1.028	0.969, 1.091	0.349
MR-PRESSO	Estimate			No outlier	-
2. Effect of T2D liability on depression					
**IVW**	**Estimate**	**39**	**1.001**	**0.989, 1.013**	**0.793**
MR-Egger	Intercept		−0.001	−0.006, 0.005	0.852
	Slope		1.005	0.964, 1.048	0.798
MR-Egger (SIMEX)	Intercept		0.001	−0.001, 0.002	0.278
	Slope		1.001	0.989, 1.013	0.808
Weighted median	Estimate		1.004	0.987, 1.022	0.581
MR-PRESSO	Estimate			No outlier	-
3. Effect of T2D liability on SCZ					
IVW	Estimate	43	1.004	0.957, 1.051	0.872
MR-Egger	Intercept		−0.003	−0.026, 0.019	0.783
	Slope		1.027	0.861, 1.226	0.758
MR-Egger (SIMEX)	Intercept		0.003	−0.003, 0.009	0.246
	Slope		1.006	0.958, 1.056	0.785
Weighted median	Estimate		0.986	0.939, 1.034	0.564
**MR-PRESSO**	**Estimate**	**41**	**0.999**	**0.993, 1.006**	**0.987**

Abbreviations: BPD: bipolar disorder; CI: confidence interval; IVW: inverse-variance weighted; MR: Mendelian randomization; MR-PRESSO: Mendelian Randomization-Pleiotropy RESidual Sum and Outlier; N: number of instruments; OR: odds of the outcome per doubling in the odds of exposure in the population (intercept of MR-Egger and MR-Egger (SIMEX) is on log‐odds scale); SCZ: schizophrenia; SIMEX: simulation extrapolation; T2D: type 2 diabetes.

The most recommended method is indicated in bold.

**TABLE 6:** Multivariable MR results for SMI liability on T2D.

MR Methods	Exposure	F-Stat	C-Q *p*	Parameter	OR	95% CI	*p*
IVW							
	BPD	30.56	-	Estimate	0.922	0.859, 0.990	0.026
	Depression	27.33	-	Estimate	1.197	1.069, 1.340	0.002
	SCZ	11.78	-	Estimate	1.030	0.980, 1.083	0.241
MR-Egger							
				Intercept	0.999	0.995, 1.003	0.769
	BPD	30.56	<0.05	Slope	0.925	0.861, 0.995	0.036
	Depression	27.33	<0.05	Slope	1.198	1.062, 1.349	0.003
	SCZ	11.78	<0.05	Slope	1.037	0.967, 1.112	0.299

Abbreviations: BPD: bipolar disorder; CI: confidence interval; C-Q *p*: Cochran’s Q-test *p*; F-Stat: F statistics; IVW: inverse-variance weighted; MR: mendelian randomization; OR: odds of the outcome per doubling in the odds of exposure in the population (intercept of MR-Egger and MR-Egger (SIMEX) is on log‐odds scale); SCZ: schizophrenia; SMI: severe mental illnesses.

The most recommended method is indicated in bold.

### Effect of liability to depression on T2D risk

Forty-two independent SNPs associated with depression liability, but not with T2D, were used as IVs. All SNP-exposure and SNP-outcome effects are presented in Supplementary Table S3. The strong instrument strength was confirmed by the *F*-statistic (37.9), and there was no measurement error of estimates from the MR study ( $I^{2}=$ 97.3%). No evidence of heterogeneity or pleiotropy was confirmed through the Q-test (*p =* 0.808), Q′-test (*p =* 0.788), and MR-PRESSO global test (*p =* 0.733) (Table 2). Since all IV assumptions were satisfied, the robust IVW method was selected to obtain promising results (OR: 1.128, 95% CI: 1.024, 1.245, *p =* 0.014) (Table 4). It was also significant even when Bonferroni correction (*p* < 0.017) controlling the family-wise error rate strictly was applied. All other sensitivity analyses showed no significant causal relationship (Table 5). In the MR for the reverse causation for T2D liability on depression, we observed that all assumptions for MR analyses were preserved (*F*-statistic = 36.0; Q-test, *p* = 0.714; Q′-test, *p* = 0.683; MR-PRESSO global test, *p* = 0.711; $I^{2}=$ 97.3%) (Table 3). Therefore, the IVW method was considered the most appropriate method and no reverse-causal effect was observed (OR: 1.001, 95% CI: 0.989, 1.013, *p* = 0.793). All other sensitivity analyses showed no significant reverse-causal relationship (Table 5). Given the sample size and $r^{2}$ , a power of 69% and 100% was estimated to detect a true OR of 1.100 for depression on T2D MR and bidirectional MR, respectively. Visualizations for bi-directional MR analysis are shown in Supplementary Figures S3 and S4. In the multivariable MR controlling for BPD and SCZ, weak instrument bias is not expected (conditional F-statistic = 27.33), and heterogeneity was detected (Cochran’s Q test *p* < 0.05). Random-effects multivariable IVW (OR: 1.197, 95% CI: 1.069, 1.340, *p =* 0.002) and multivariable MR-Egger (OR: 1.198, 95% CI: 1.062, 1.349, *p =* 0.003) provide a corrected causal estimate when such pleiotropy exists (Table 6).

### Effect of liability to SCZ on T2D risk

Seventy-two independent SNPs associated with SCZ liability, rather than T2D, were found to have strong instrument strength (*F*-statistic = 41.5) and no heterogeneity and outlier pleiotropy (Q-test, *p =* 0.319; Q′-test, *p =* 0.290; MR-PRESSO global test, *p =* 0.704) (Table 3). All SNP-exposure and SNP-outcome effects are shown in Supplementary Table S3. In addition, there was no dilution bias from violation of the NOME assumption ( $I^{2}=$ 97.2%). The IVW method was the most powerful method, and it showed no causal effect of SCZ liability on T2D (OR: 1.011, 95% CI: 0.981, 1.040, *p =* 0.463). Results of sensitivity MR analysis showed no evidence about the SCZ liability on T2D with wide CIs (Table 4). In the MR for the reverse causation for T2D liability on SCZ, *F*-statistic showed no weak instrument bias (*F*-statistic = 35.8) or violation of the NOME assumption ( $I^{2}=$ 97.2) (Table 3). The heterogeneity test showed substantial evidence of outlier pleiotropy using the Q-test (*p* < 0.001), Q′-test (*p* < 0.001), and MR-PRESSO global test (*p* < 0.001) (Table 3). Since all the three tests were rejected, the MR-PRESSO method was adopted (Jin et al., 2020) after excluding two outlier SNPs (OR: 0.999, 95% CI: 0.993, 1.006, *p =* 0.987), suggesting no causal effect of T2D liability on SCZ. Given the sample size and $r^{2}$ , we were 69% and 89.5% powered to detect a true OR of 1.100 for both SCZ on T2D MR and bidirectional MR. Visualizations for bi-directional MR analysis are shown in Supplementary Figures S5 and S6. In the multivariable MR controlling for BPD and depression, weak instrument bias is not expected (conditional F-statistic = 11.78), and heterogeneity was detected (Cochran’s Q test *p* < 0.05). Random-effects multivariable IVW and multivariable MR-Egger results showed that the effect of SCZ liability did not have a causal effect on T2D (Table 6).

## Discussion

In this study, we showed genetic correlations between depression and SCZ with T2D, while no genetic association was found between BPD and T2D from LDSC regression. Two-sample univariable and multivariable MR results provided some evidence in support of the hypothesis that depression liability increases the risk of T2D, whereas there was no evidence to suggest that liability to BPD and SCZ are risk factors for T2D. Additionally, a bidirectional MR study found no reverse causality between SMI and T2D, which supports the hypothesis that depression is one of the causal factors influencing T2D. However, observational studies have provided contradictory and controversial findings. One systematic review demonstrated that depression is associated with a 60% increased risk of T2D, while the evidence is also compatible with the high prevalence rates of depression among individuals with T2D (Lustman et al., 1986). A large meta-analysis showed that T2D is associated with only a modestly increased risk of depression (Mezuk et al., 2008). Depression is difficult to detect in older adults, which may partially explain the utter modesty of this association (Gallo et al., 1994). On the other hand, a MR study based on East Asian populations demonstrated that T2D as a chronic disorder would increase the risk of depression (Xuan et al., 2018). It is important to note that the causal relationship between depression and T2D may vary depending on race or ethnicity, as indicated by the contrasting results in different populations. In addition, the difference in statistical power due to sample size could have led to the discrepancy in the results.

Our finding, which relates to the causal role of depression liability in an increased risk of T2D, could be explained by the pathophysiological mechanisms underlying the two diseases. Two major molecular mechanisms have been suggested to explain the causal pathway between them. First, the hypothalamic-pituitary-adrenal axis, a central stress response system, is commonly activated in patients with depression suffering from emotional stressors leading to a rise in the levels of glucocorticoids, primarily cortisol (Tsigos and Chrousos, 2002). High cortisol level induces and aggravates insulin resistance in a vicious cycle (Geer et al., 2014). Second, sympathetic nervous system (SNS) activity is also elevated in depression (Tabák et al., 2004). The SNS axis interacts with the hypothalamic-pituitary-adrenal axis to maintain homeostasis during stress, resulting in an increased release of cortisol and other glucocorticoids, catecholamines, growth hormone, and glucagon. Indeed, catecholamines have marked metabolic effects, particularly on glucose metabolism (Barth et al., 2007).

However, our findings are inconsistent with an observational study suggesting a causal role of liability to BPD and SCZ in the risk of T2D and that liability to T2D predicts the development of depression (Regenold et al., 2002). Such associations may have been driven by residual confounders, and several suggestive factors can act as confounders. First, a sedentary lifestyle, demonstrated to be strongly associated with SMI, may play a role as a potential confounder (Vancampfort et al., 2016). A large meta‐analysis of general population studies reported that sedentary behavior is independently associated with an increased risk of T2D (Biswas et al., 2015). Additionally, the side effects of medication could be another important potential confounder. A systematic review of cross-sectional and prospective studies indicated that the use of antipsychotics, antidepressants, and mood stabilizers could contribute to an increased body mass index, which is a major risk factor for T2D (Correll et al., 2015). Furthermore, the highest prevalence of daily cigarette smoking was observed among patients with SCZ, followed by patients with BPD and those with depression, compared with the general population. The association with smoking is stronger in SCZ and BPD than in depression (Diaz et al., 2009). The evidence that nicotine addiction begins before any of these SMIs develop suggests the involvement of shared genes associated with nicotine addiction and SMI (Kendler et al., 1993). In contrast, in MR analysis, genetic variants (i.e., SNPs) used as IVs are innately random, and are assumed to be independent of confounding factors such as age, gender, and race.

MR studies on the association between SMI liability and T2D are scarce, with no studies on liability to BPD comorbid with T2D. To investigate the potential causal relationship of T2D with depression, MR analysis was performed with a large Chinese longitudinal cohort from 2011 to 2013 (Xuan et al., 2018). In their studies, effect of depression on T2D was not significant, which is inconsistent with our finding. There are multiple reasons about such inconsistency. First, there may be a racial difference between non-Hispanic whites and Chinese. Second, we considered two-stage methods and Xuan et al. considered one-sample Mendelian randomizations. Both methods require several assumptions to extend the analysis results to the causality of depression on T2D, and if they are not satisfied, causality cannot be guaranteed. For instance, some of assumptions such as horizontal pleiotropy can be violated. Furthermore, the fitted values from the first-stage regression are correlated with the outcome in finite samples even, and there can be a finite-sample bias in a one-sample setting (Gallo et al., 1994). Regarding the MR studies of SCZ and T2D, two-sample MR was performed using the IVW and MR-Egger methods in European, East Asian, and trans-ancestry groups (Burgess et al., 2016b). No evidence of a causal effect on T2D for SCZ was observed in any analyses, consistent with our findings; however, they did not perform a bidirectional analysis to investigate the causal effect of SCZ on T2D. On the other hand, a study demonstrated that SCZ can be considered as a causal factor for T2D, which contrasts with the findings of our research (Cai et al., 2022). In MR studies, the selection of instruments is crucial, as the choice of IVs (i.e., SNPs) can lead to different results. However, this study and our research not only used different GWAS summary statistics for T2D but also had only 37 common SNPs, which accounted for approximately 50% of the IVs in both studies. Therefore, the differences in the results between both studies can be attributed to disparities in the analysis samples and IVs. Unlike epidemiological studies, previous and present MR studies could not consider the multi‐episode status of the disease, which may have led to the non-causal effect of SCZ and BPD. This could be because multi‐episode (*versus* first‐episode) patients with SMI were more likely to have T2D than matched controls in the meta‐analysis of observational studies (Vancampfort et al., 2016).

Our study has some limitations. First, our research has a potential limitation for “winner’s curse” in a two-sample MR framework using SNPs as instruments from discovery GWASs, which can cause bias. Second, there are significant genetic correlation between SCZ with T2D but there is no evidence of causality. This indicates that the observed association could potentially be influenced by other factors such as population stratification or sample overlapping between the two GWAS. Therefore, further studies with designs that are not influenced by these factors are needed. Third, there are different clinical subtypes of depression (melancholic, psychotic, atypical, or undifferentiated), BPD (type 1 or 2), and mood states (manic, depressive, mixed, or euthymic); however, a large category of diseases was analyzed without distinction. A mixture of classifications is problematic because the effect of the subtype disease liability on T2D may differ even if they are included in the same SMI category. Especially in the case of BPD or SCZ liability, the causal effect on T2D may have been annulled depending on the diseases’ subtype. Fourth, although we conducted bidirectional MR studies, the sample size of GWASs for BPD (<100,000) was relatively small, which could lead to low analysis power (50.8%) with a true OR of less than 1.100. The reliability of the analysis result is low, and further MR studies with large sample size are required. Fifth, we only included a European population; hence, it is difficult to apply the same clinical interpretation to other populations. Finally, horizontal pleiotropy, a natural flaw of MR design, can occurs when a genetic variant affects the outcome variable without mediating the exposure variable (Chen et al., 2022).

## Conclusion

This study provided evidence for depression liability having a causal effect on T2D, which is supported by previously reported biological mechanisms. Therefore, it is imperative to consider screening for diabetes and metabolic abnormalities in patients with depression or probable depression.

## Data Availability

Publicly available datasets were analyzed in this study. The datasets generated and/or analyzed during the current study are available in the DIAbetes Genetics Replication and Meta-analysis Consortium repository, https://www.diagram-consortium.org/downloads.html (SHA1:d48402f1ce501ea5b98335e6e15173bb865bb6f1, MD5:f033ebb95e01afedde7365e4fc6565a2) and Psychiatric Genomics Consortium repository, https://figshare.com/articles/dataset/bip2019/14671998 (BPD), https://datashare.ed.ac.uk/handle/10283/3203 (depression), and https://figshare.com/articles/dataset/scz2019asi/19193084 (SCZ).

## Abbreviations

BPD, bipolar disorder; CI, confidence interval; GWAS, genome-wide association study; ICD, International Classification of Diseases; IVW, inverse-variance weighted; LD, linkage disequilibrium; MR, Mendelian randomization; MR-PRESSO, Mendelian Randomization-Pleiotropy RESidual Sum and Outlier; NOME, NO Measurement Error; OR, odds ratio; SCZ, schizophrenia; SMI, severe mental illness; SIMEX, simulation extrapolation; SNP, single nucleotide polymorphism; SNS, sympathetic nervous system; T2D, type 2 diabetes.
