# Supplementary material for: Mendelian randomization shows depression increases the risk of type 2 diabetes
Source: Front Genet. 2023 Aug 24;14:1181851. doi: 10.3389/fgene.2023.1181851 (PMC10484410; doi:10.3389/fgene.2023.1181851)
Supplement: Supplementary file 1 [file DataSheet2.docx]

**Supplementary Figure 1.** MR analysis visualizations of the effect of BPD on T2D


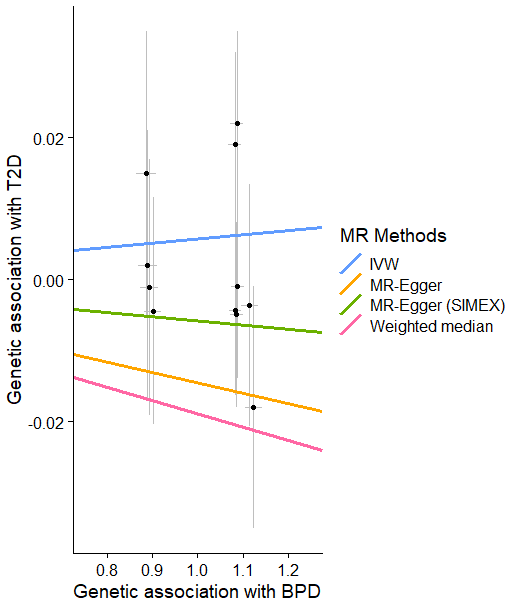

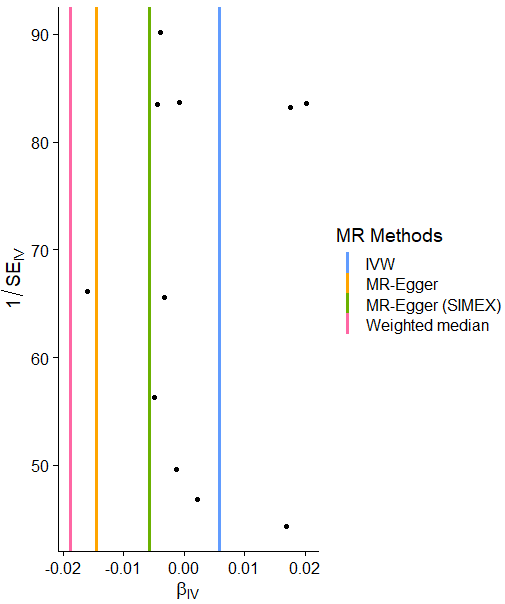
 **(a) (b)**

**(c) (d)**


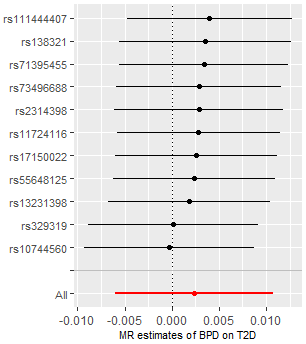

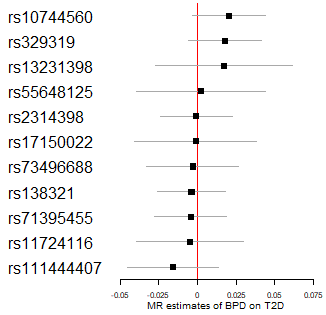


**(a)** Scatter plot showing the associations of the genetic variants with BPD liability on T2D for different methods of MR. **(b)** Funnel plot displaying the relationship between the causal effect of BPD on T2D estimated by each SNP against the inverse of the standard error of the causal estimate. Vertical lines show the MR estimates for different methods of MR. **(c)** Forest plot representing the causal estimate of BPD (SD units) on T2D **(d)** Leave-one-out sensitivity analysis representing the maximum likelihood MR method applied to estimate the causal effect of BPD on T2D excluding the target variant from the analysis. The red point means the estimate obtained using the IVW method.

Abbreviations: BPD = bipolar disorder, T2D = type 2 diabetes, MR = Mendelian randomization, IVW = inverse variance weighted, SIMEX = simulation extrapolation, IV = instrumental variable

**Supplementary Figure 2.** Bidirectional MR analysis visualizations of the effect of T2D on BPD

1. **(b)**


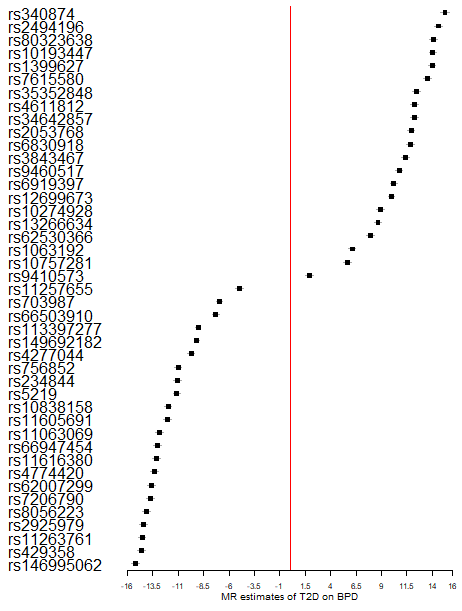

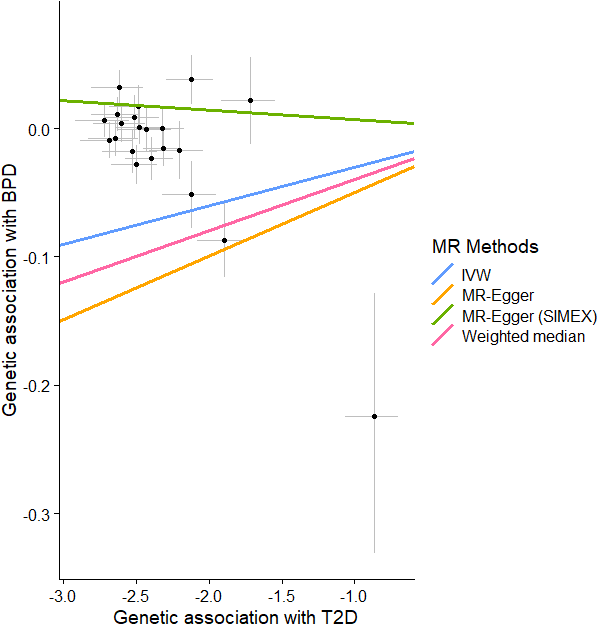

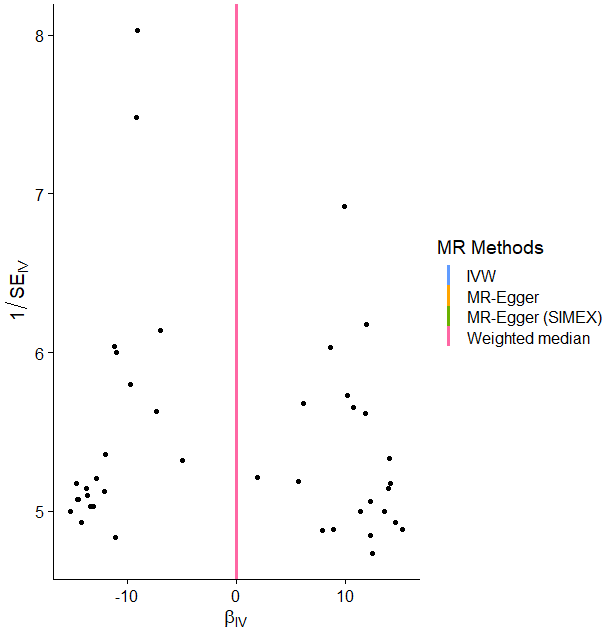
 **(c) (d)**


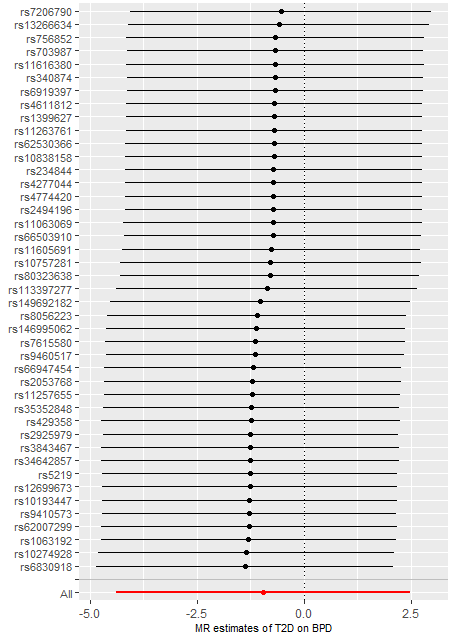


**(a)** Scatter plot showing the associations of the genetic variants with T2D liability on BPD for different methods of MR. **(b)** Funnel plot displaying the relationship between the causal effect of T2D on BPD estimated by each SNP against the inverse of the standard error of the causal estimate. Vertical lines show the MR estimates for different methods of MR. **(c)** Forest plot representing the causal estimate of T2D (SD units) on BPD **(d)** Leave-one-out sensitivity analysis representing the maximum likelihood MR method applied to estimate the causal effect of T2D on BPD excluding the target variant from the analysis. The red point means the estimate obtained using the IVW method.

Abbreviations: BPD = bipolar disorder, T2D = type 2 diabetes, MR = Mendelian randomization, IVW = inverse variance weighted, SIMEX = simulation extrapolation, IV = instrumental variable

**Supplementary Figure 3.** MR analysis visualizations of the effect of depression on T2D


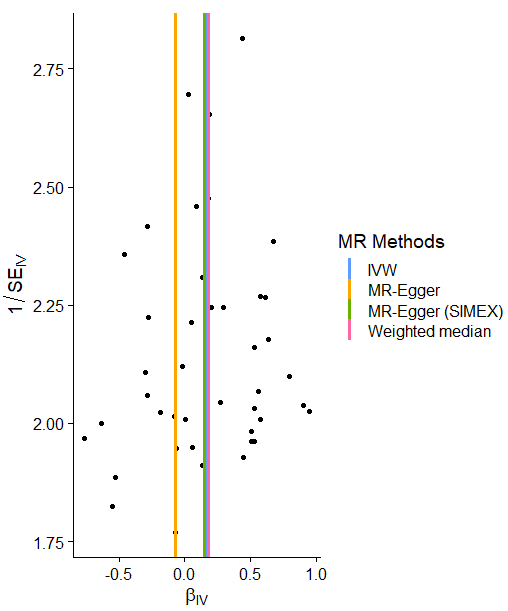

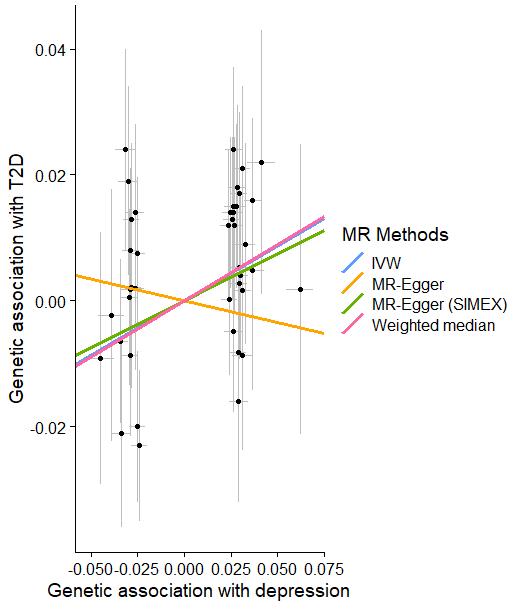
**(a) (b)**

**(c) (d)**


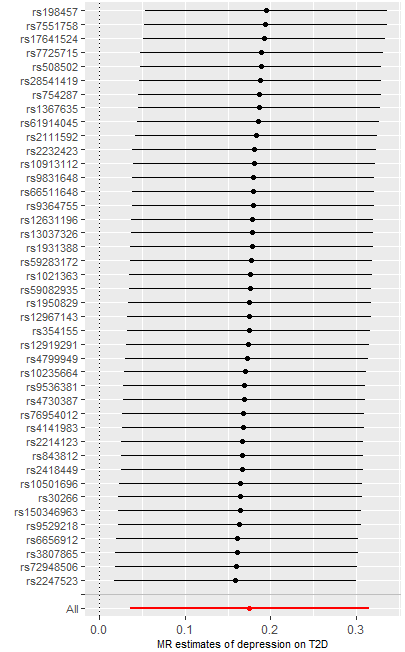

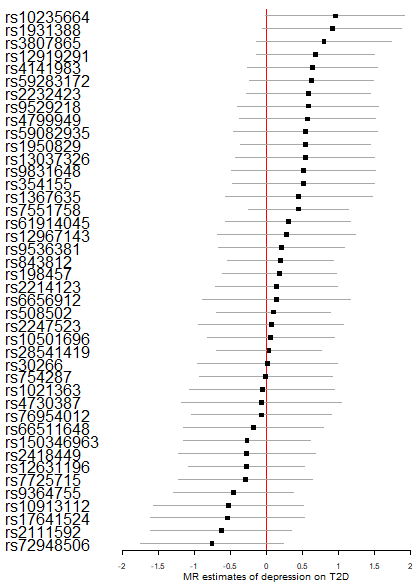


**(a)** Scatter plot showing the associations of the genetic variants with depression liability on T2D for different methods of MR. **(b)** Funnel plot displaying the relationship between the causal effect of depression on T2D estimated by each SNP against the inverse of the standard error of the causal estimate. Vertical lines show the MR estimates for different methods of MR. **(c)** Forest plot representing the causal estimate of depression (SD units) on T2D **(d)** Leave-one-out sensitivity analysis representing the maximum likelihood MR method applied to estimate the causal effect of depression on T2D excluding the target variant from the analysis. The red point means the estimate obtained using the IVW method.

Abbreviations: T2D = type 2 diabetes, MR = Mendelian randomization, IVW = inverse variance weighted, SIMEX = simulation extrapolation, IV = instrumental variable

**Supplementary Figure 4.** Bidirectional MR analysis visualizations of the effect of T2D on depression

1.
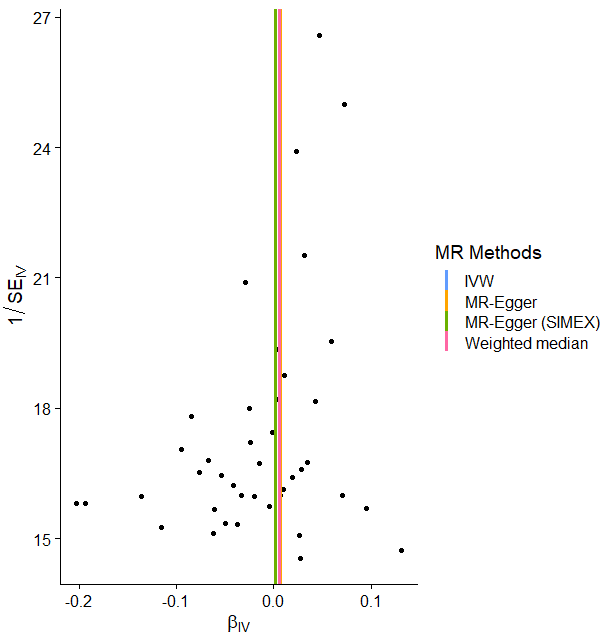

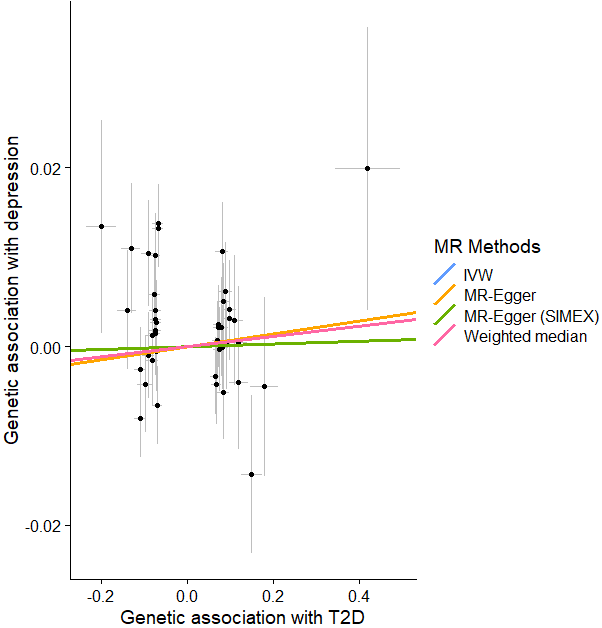
 **(b)**


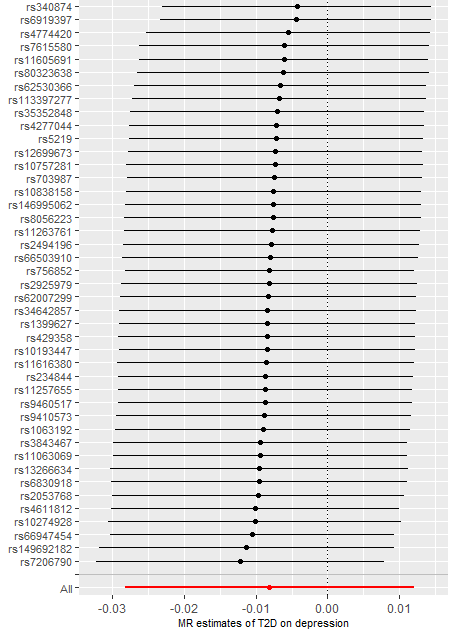

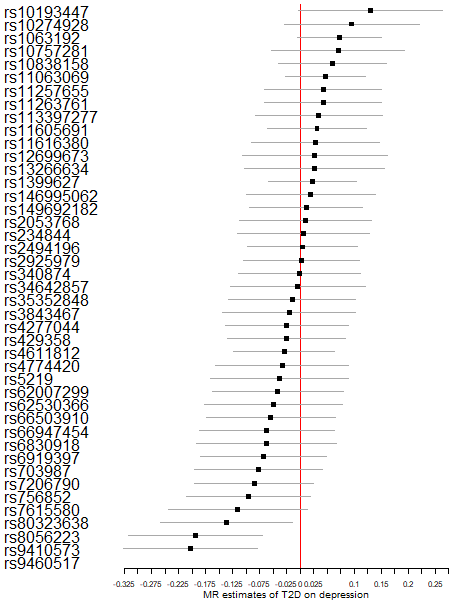
  **(c) (d)**

**(a)** Scatter plot showing the associations of the genetic variants with T2D liability on depression for different methods of MR. **(b)** Funnel plot displaying the relationship between the causal effect of T2D on depression estimated by each SNP against the inverse of the standard error of the causal estimate. Vertical lines show the MR estimates for different methods of MR. **(c)** Forest plot representing the causal estimate of T2D (SD units) on depression **(d)** Leave-one-out sensitivity analysis representing the maximum likelihood MR method applied to estimate the causal effect of T2D on depression excluding the target variant from the analysis. The red point means the estimate obtained using the IVW method.

Abbreviations: T2D = type 2 diabetes, MR = Mendelian randomization, IVW = inverse variance weighted, SIMEX = simulation extrapolation, IV = instrumental variable

**Supplementary Figure 5.** MR analysis visualizations of the effect of SCZ on T2D


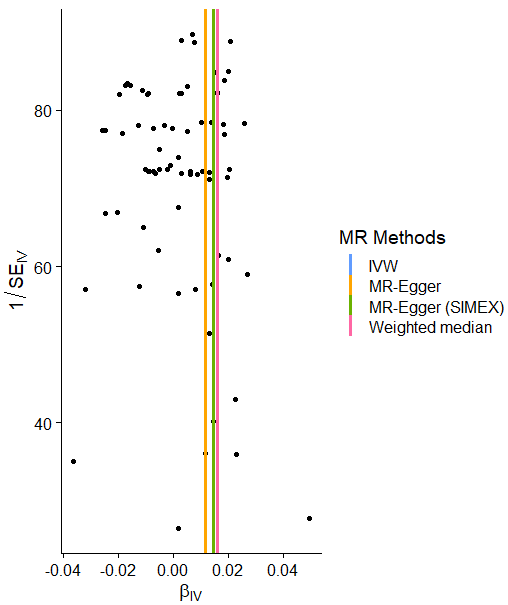

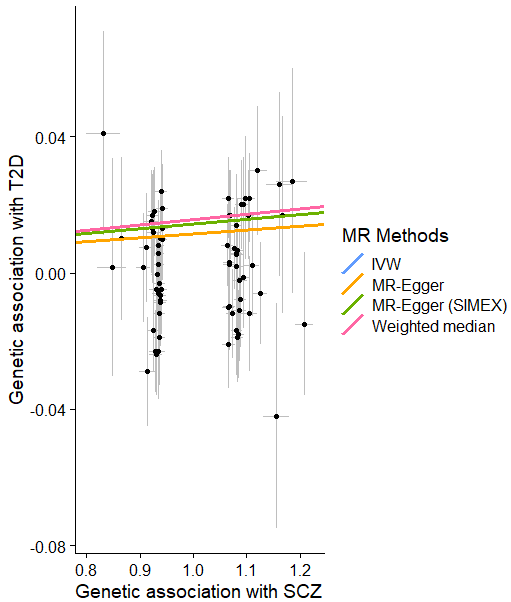
**(a) (b)**


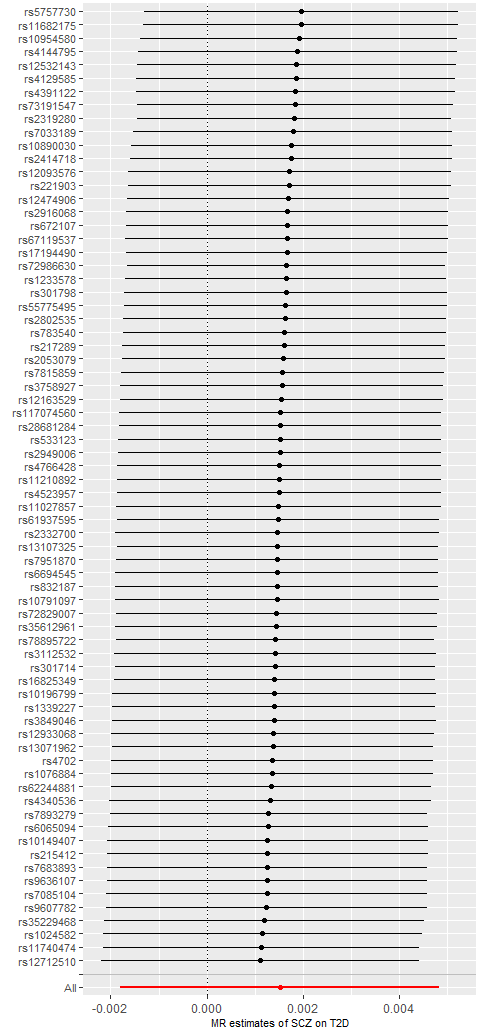

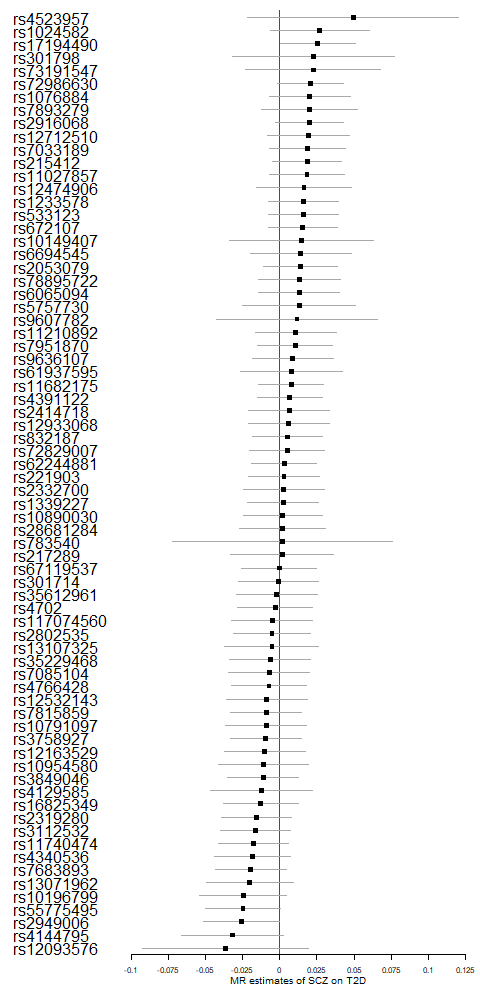
**(c) (d)**

**(a)** Scatter plot showing the associations of the genetic variants with SCZ liability on T2D for different methods of MR. **(b)** Funnel plot displaying the relationship between the causal effect of SCZ on T2D estimated by each SNP against the inverse of the standard error of the causal estimate. Vertical lines show the MR estimates for different methods of MR. **(c)** Forest plot representing the causal estimate of SCZ (SD units) on T2D **(d)** Leave-one-out sensitivity analysis representing the maximum likelihood MR method applied to estimate the causal effect of SCZ on T2D excluding the target variant from the analysis. The red point means the estimate obtained using the IVW method.

Abbreviations: SCZ = schizophrenia, T2D = type 2 diabetes, MR = Mendelian randomization, IVW = inverse variance weighted, SIMEX = simulation extrapolation, IV = instrumental variable


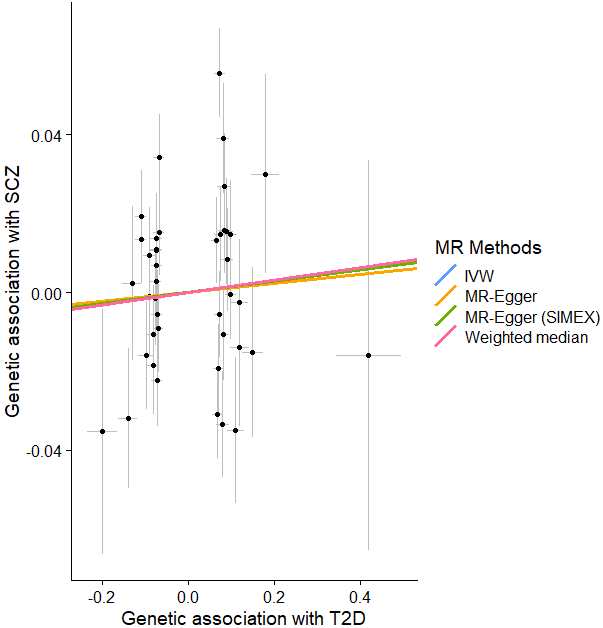
**Supplementary Figure 6.** Bidirectional MR analysis visualizations of the effect of T2D on SCZ

1.
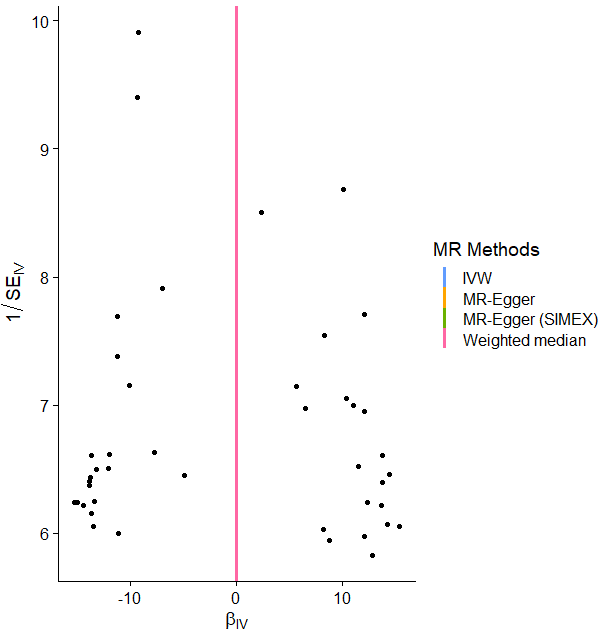
**(b)**


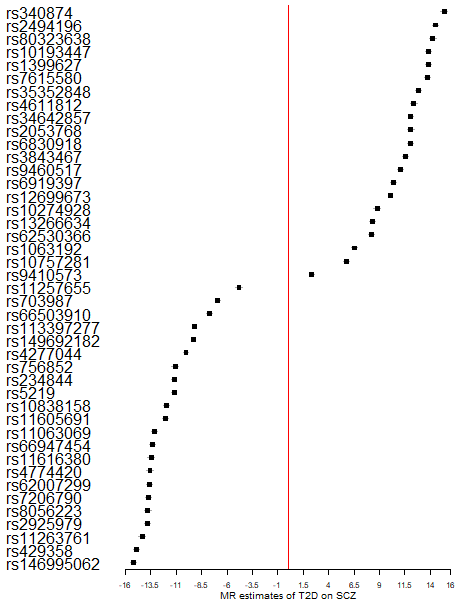

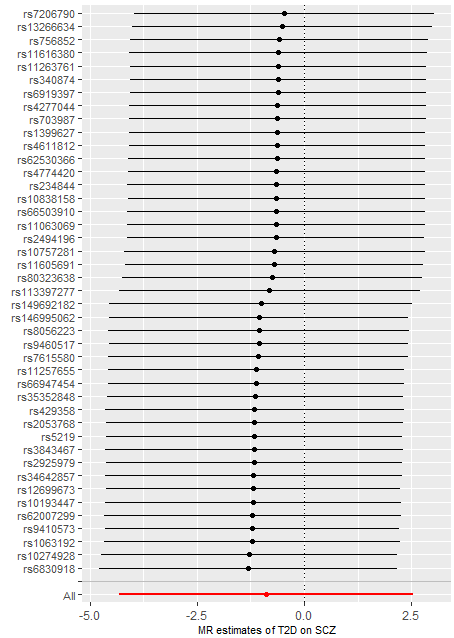
 **(c) (d)**

**(a)** Scatter plot showing the associations of the genetic variants with T2D liability on SCZ for different methods of MR. **(b)** Funnel plot displaying the relationship between the causal effect of T2D on SCZ estimated by each SNP against the inverse of the standard error of the causal estimate. Vertical lines show the MR estimates for different methods of MR. **(c)** Forest plot representing the causal estimate of T2D (SD units) on SCZ **(d)** Leave-one-out sensitivity analysis representing the maximum likelihood MR method applied to estimate the causal effect of T2D on SCZ excluding the target variant from the analysis. The red point means the estimate obtained using the IVW method.

Abbreviations: SCZ = schizophrenia, T2D = type 2 diabetes, MR = Mendelian randomization, IVW = inverse variance weighted, SIMEX = simulation extrapolation, IV = instrumental variable

**Supplementary Figure 7.** Stacked Manhattan plots for BPD, depression, SCZ, and T2D


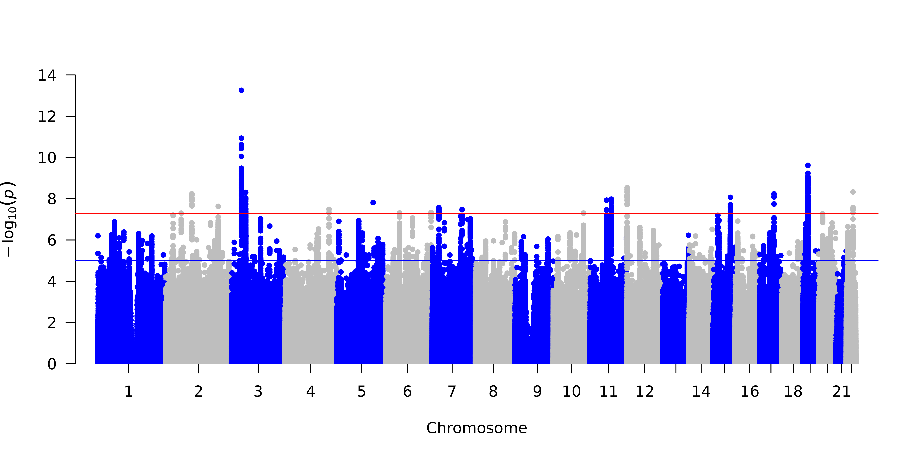

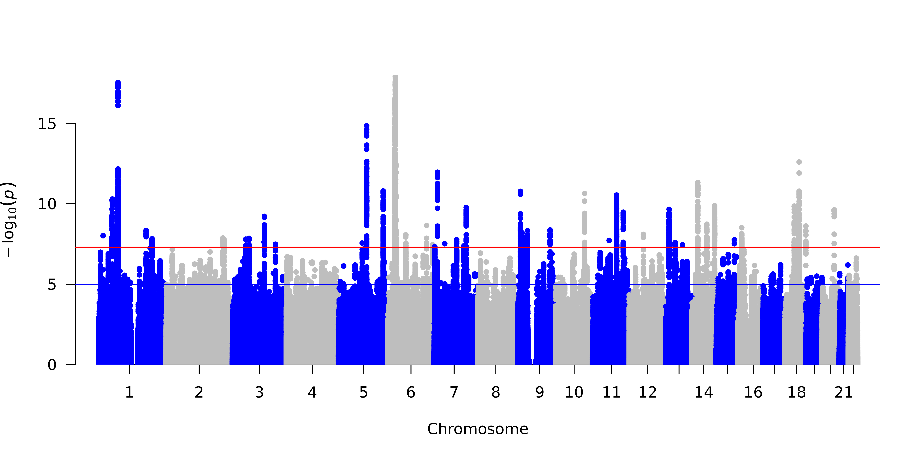

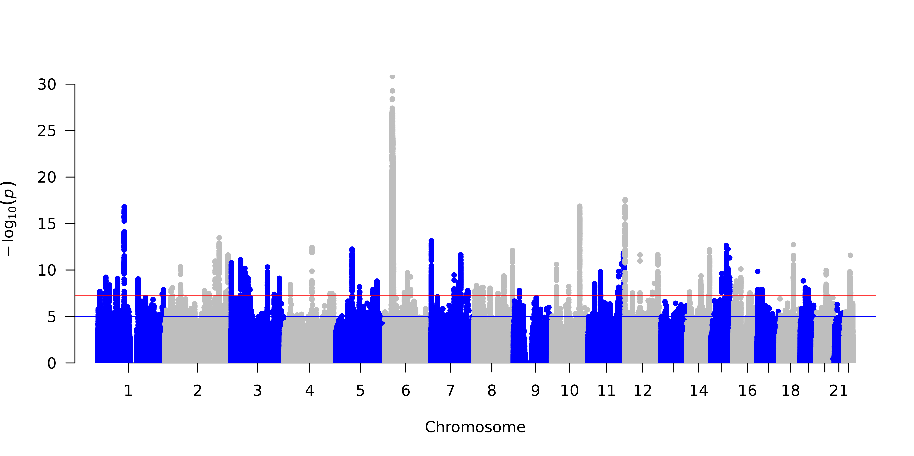

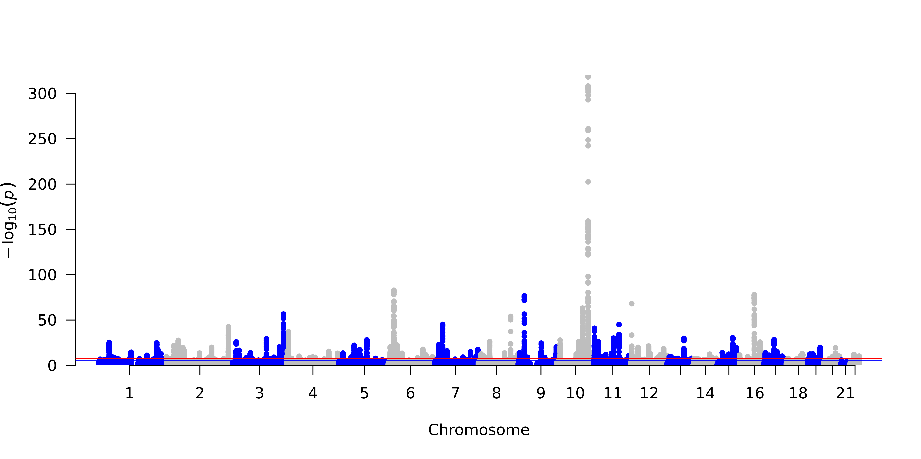


BPD

Depression

SCZ

T2D

Stacked Manhattan plots of the P-values in the genome-wide association study for BPD, depression, SCZ, and T2D (red = genome-wide line, blue = suggestive line).
