# Supplementary material for: Mendelian randomization shows depression increases the risk of type 2 diabetes
Source: Front Genet. 2023 Aug 24;14:1181851. doi: 10.3389/fgene.2023.1181851 (PMC10484410; doi:10.3389/fgene.2023.1181851)
Supplement: Supplementary file 2 [file DataSheet1.docx]

| Chr | SNP | Allele^a^ | SNP-Exposure | | | SNP-Outcome | | |
| --- | --- | --- | --- | --- | --- | --- | --- | --- |
|  |  |  | LogOR^b^ | SE^c^ | *P-value* | LogOR^b^ | SE^c^ | *P-value* |
| 2 | rs2314398 | C/G | 1.087 | 0.014 | 5.92${\times10}^{-9}$ | -0.0009 | 0.013 | 0.940 |
| 4 | rs11724116 | T/C | 0.901 | 0.019 | 3.27${\times10}^{-8}$ | -0.0044 | 0.016 | 0.790 |
| 5 | rs329319 | A/G | 1.081 | 0.014 | 1.54${\times10}^{-8}$ | 0.019 | 0.013 | 0.140 |
| 6 | rs55648125 | A/G | 0.889 | 0.022 | 4.92${\times10}^{-8}$ | 0.002 | 0.019 | 0.920 |
| 7 | rs17150022 | T/C | 0.892 | 0.020 | 2.70${\times10}^{-8}$ | -0.0011 | 0.018 | 0.950 |
| 7 | rs13231398 | C/G | 0.886 | 0.022 | 3.36${\times10}^{-8}$ | 0.015 | 0.020 | 0.450 |
| 11 | rs73496688 | A/T | 1.114 | 0.019 | 1.05${\times10}^{-8}$ | -0.0036 | 0.017 | 0.840 |
| 12 | rs10744560 | T/C | 1.086 | 0.014 | 2.92${\times10}^{-9}$ | 0.022 | 0.013 | 0.095 |
| 15 | rs71395455 | A/G | 1.085 | 0.015 | 1.93${\times10}^{-8}$ | -0.0049 | 0.013 | 0.710 |
| 19 | rs111444407 | T/C | 1.123 | 0.018 | 2.40${\times10}^{-10}$ | -0.018 | 0.017 | 0.300 |
| 22 | rs138321 | A/G | 1.082 | 0.014 | 4.69${\times10}^{-9}$ | -0.0043 | 0.012 | 0.720 |

**Supplementary Table 1.** Instrument variables for BPD and T2D

^a^Allele: risk/reference allele; ^b^LogOR: log odds ratio; ^c^SE: standard error

BPD: bipolar disorder, Chr: chromosome, SNP: single nucleotide polymorphism, T2D: type 2 diabetes

| Chr | SNP | Allele^a^ | SNP-Exposure | | | SNP-Outcome | | |
| --- | --- | --- | --- | --- | --- | --- | --- | --- |
|  |  |  | LogOR^b^ | SE^c^ | *P-value* | LogOR^b^ | SE^c^ | *P-value* |
| 1 | rs4141983 | T/C | 0.026 | 0.005 | 9.69$\times{10}^{-9}$ | 0.014 | 0.013 | 0.280 |
| 1 | rs354155 | C/G | -0.045 | 0.008 | 1.75$\times{10}^{-9}$ | -0.009 | 0.020 | 0.640 |
| 1 | rs7551758 | T/G | -0.028 | 0.004 | 5.11$\times{10}^{-11}$ | 0.013 | 0.012 | 0.270 |
| 1 | rs6656912 | T/C | -0.025 | 0.004 | 6.50$\times{10}^{-9}$ | -0.020 | 0.012 | 0.100 |
| 1 | rs10913112 | T/C | -0.026 | 0.005 | 4.53$\times{10}^{-9}$ | 0.002 | 0.013 | 0.880 |
| 1 | rs17641524 | T/C | -0.030 | 0.005 | 1.50$\times{10}^{-8}$ | 0.019 | 0.015 | 0.180 |
| 2 | rs2111592 | A/G | 0.026 | 0.005 | 1.35$\times{10}^{-8}$ | -0.005 | 0.013 | 0.720 |
| 2 | rs72948506 | A/G | 0.027 | 0.005 | 1.72$\times{10}^{-8}$ | 0.024 | 0.013 | 0.072 |
| 3 | rs9831648 | T/G | -0.029 | 0.005 | 1.59$\times{10}^{-8}$ | 0.002 | 0.015 | 0.900 |
| 3 | rs843812 | A/G | 0.025 | 0.004 | 1.41$\times{10}^{-8}$ | 0.014 | 0.012 | 0.240 |
| 3 | rs76954012 | A/T | 0.041 | 0.007 | 2.41$\times{10}^{-8}$ | 0.022 | 0.021 | 0.300 |
| 3 | rs66511648 | T/C | -0.030 | 0.005 | 6.03$\times{10}^{-10}$ | 0.001 | 0.014 | 0.970 |
| 3 | rs12631196 | A/G | 0.024 | 0.004 | 3.28$\times{10}^{-8}$ | 0.000 | 0.012 | 0.990 |
| 5 | rs30266 | A/G | 0.037 | 0.005 | 1.43$\times{10}^{-15}$ | 0.016 | 0.013 | 0.230 |
| 5 | rs7725715 | A/G | 0.029 | 0.004 | 1.61$\times{10}^{-11}$ | -0.008 | 0.012 | 0.500 |
| 6 | rs2232423 | A/G | 0.062 | 0.007 | 1.14$\times{10}^{-18}$ | 0.002 | 0.023 | 0.940 |
| 6 | rs2214123 | A/G | 0.026 | 0.005 | 8.56$\times{10}^{-9}$ | 0.015 | 0.013 | 0.230 |
| 6 | rs9364755 | A/G | -0.028 | 0.005 | 3.49$\times{10}^{-8}$ | 0.002 | 0.016 | 0.900 |
| 7 | rs10235664 | T/C | 0.027 | 0.005 | 4.68$\times{10}^{-8}$ | 0.012 | 0.014 | 0.380 |
| 7 | rs3807865 | A/G | 0.031 | 0.004 | 1.09$\times{10}^{-12}$ | 0.021 | 0.013 | 0.093 |
| 7 | rs59082935 | T/C | 0.036 | 0.007 | 3.07$\times{10}^{-8}$ | 0.005 | 0.019 | 0.800 |
| 7 | rs2247523 | C/G | -0.024 | 0.004 | 1.71$\times{10}^{-8}$ | -0.023 | 0.012 | 0.052 |
| 7 | rs4730387 | A/T | 0.024 | 0.004 | 4.12$\times{10}^{-8}$ | 0.012 | 0.012 | 0.310 |
| 7 | rs150346963 | T/C | 0.028 | 0.004 | 1.16$\times{10}^{-10}$ | 0.018 | 0.013 | 0.140 |
| 9 | rs1931388 | A/G | 0.030 | 0.004 | 1.68$\times{10}^{-11}$ | 0.003 | 0.012 | 0.830 |
| 9 | rs59283172 | A/G | -0.039 | 0.007 | 2.41$\times{10}^{-8}$ | -0.002 | 0.020 | 0.910 |
| 9 | rs2418449 | T/C | 0.028 | 0.005 | 4.25$\times{10}^{-9}$ | 0.015 | 0.013 | 0.270 |
| 10 | rs1021363 | A/G | 0.030 | 0.005 | 2.29$\times{10}^{-11}$ | 0.004 | 0.013 | 0.750 |
| 11 | rs198457 | T/C | -0.032 | 0.006 | 1.90$\times{10}^{-8}$ | 0.024 | 0.016 | 0.130 |
| 11 | rs10501696 | A/G | 0.030 | 0.004 | 2.89$\times{10}^{-11}$ | 0.017 | 0.013 | 0.200 |
| 12 | rs61914045 | A/G | 0.031 | 0.005 | 7.96$\times{10}^{-9}$ | -0.009 | 0.015 | 0.560 |
| 13 | rs9529218 | T/C | -0.034 | 0.005 | 2.23$\times{10}^{-10}$ | -0.021 | 0.015 | 0.170 |
| 13 | rs9536381 | T/C | 0.026 | 0.005 | 2.62$\times{10}^{-8}$ | 0.013 | 0.013 | 0.320 |
| 13 | rs508502 | T/C | -0.026 | 0.005 | 3.56$\times{10}^{-8}$ | 0.014 | 0.014 | 0.310 |
| 14 | rs1950829 | A/G | 0.030 | 0.004 | 4.74$\times{10}^{-12}$ | 0.005 | 0.012 | 0.660 |
| 14 | rs754287 | A/T | -0.029 | 0.005 | 1.31$\times{10}^{-10}$ | 0.008 | 0.013 | 0.530 |
| 15 | rs28541419 | C/G | 0.029 | 0.005 | 1.76$\times{10}^{-8}$ | -0.016 | 0.016 | 0.300 |
| 16 | rs12919291 | C/G | 0.033 | 0.006 | 3.09$\times{10}^{-9}$ | 0.009 | 0.016 | 0.570 |
| 18 | rs4799949 | T/C | -0.029 | 0.005 | 1.40$\times{10}^{-10}$ | -0.009 | 0.013 | 0.510 |
| 18 | rs1367635 | T/C | -0.025 | 0.004 | 4.35$\times{10}^{-9}$ | 0.008 | 0.012 | 0.530 |
| 18 | rs12967143 | CG | -0.035 | 0.005 | 2.53$\times{10}^{-13}$ | -0.006 | 0.013 | 0.640 |
| 20 | rs13037326 | T/C | 0.031 | 0.005 | 2.40$\times{10}^{-10}$ | 0.002 | 0.014 | 0.900 |

**Supplementary Table 2.** Instrument variables for depression and T2D

^a^Allele: risk/reference allele; ^b^LogOR: log odds ratio; ^c^SE: standard error

Chr: chromosome, SNP: single nucleotide polymorphism, T2D: type 2 diabetes

**Supplementary Table 3.** Instrument variables for SCZ and T2D

| Chr | SNP | Allele^a^ | SNP-Exposure | | | SNP-Outcome | | |
| --- | --- | --- | --- | --- | --- | --- | --- | --- |
|  |  |  | LogOR^b^ | SE^c^ | *P-value* | LogOR^b^ | SE^c^ | *P-value* |
| 1 | rs301798 | A/G | 0.937 | 0.012 | 2.04$\times{10}^{-8}$ | -0.007 | 0.013 | 0.600 |
| 1 | rs533123 | A/G | 1.081 | 0.014 | 3.64$\times{10}^{-8}$ | 0.002 | 0.016 | 0.900 |
| 1 | rs6694545 | A/G | 1.082 | 0.013 | 6.06$\times{10}^{-8}$ | 0.007 | 0.015 | 0.650 |
| 1 | rs11210892 | A/G | 0.935 | 0.012 | 4.13$\times{10}^{-8}$ | 0.003 | 0.013 | 0.850 |
| 1 | rs10890030 | T/C | 0.936 | 0.011 | 1.22$\times{10}^{-9}$ | -0.012 | 0.012 | 0.330 |
| 1 | rs2802535 | T/C | 1.125 | 0.014 | 1.61$\times{10}^{-17}$ | -0.006 | 0.015 | 0.690 |
| 1 | rs2319280 | A/C | 0.913 | 0.015 | 9.61$\times{10}^{-10}$ | -0.029 | 0.016 | 0.075 |
| 1 | rs12093576 | T/C | 1.065 | 0.011 | 1.31$\times{10}^{-8}$ | -0.010 | 0.013 | 0.430 |
| 2 | rs12712510 | T/C | 1.065 | 0.011 | 2.38$\times{10}^{-8}$ | 0.022 | 0.012 | 0.075 |
| 2 | rs12474906 | A/C | 1.085 | 0.014 | 7.98$\times{10}^{-9}$ | -0.011 | 0.015 | 0.480 |
| 2 | rs11682175 | T/C | 0.929 | 0.011 | 4.61$\times{10}^{-11}$ | -0.023 | 0.012 | 0.056 |
| 2 | rs16825349 | A/G | 0.923 | 0.014 | 1.84$\times{10}^{-8}$ | 0.013 | 0.016 | 0.420 |
| 2 | rs4340536 | C/G | 0.942 | 0.011 | 3.57$\times{10}^{-8}$ | 0.013 | 0.012 | 0.300 |
| 2 | rs10196799 | A/T | 1.077 | 0.011 | 1.12$\times{10}^{-11}$ | 0.007 | 0.012 | 0.550 |
| 2 | rs55775495 | T/C | 0.934 | 0.011 | 1.91$\times{10}^{-9}$ | -0.006 | 0.013 | 0.630 |
| 2 | rs2949006 | T/G | 1.110 | 0.014 | 3.45$\times{10}^{-14}$ | 0.002 | 0.015 | 0.890 |
| 2 | rs4144795 | C/G | 1.082 | 0.011 | 3.12$\times{10}^{-12}$ | -0.019 | 0.013 | 0.150 |
| 3 | rs17194490 | T/G | 1.104 | 0.015 | 1.69$\times{10}^{-11}$ | -0.012 | 0.017 | 0.470 |
| 3 | rs832187 | T/C | 0.933 | 0.011 | 7.33$\times{10}^{-10}$ | 0.006 | 0.013 | 0.650 |
| 3 | rs62244881 | T/C | 1.097 | 0.016 | 1.29$\times{10}^{-8}$ | 0.022 | 0.018 | 0.210 |
| 3 | rs12163529 | A/G | 0.932 | 0.011 | 1.59$\times{10}^{-10}$ | 0.000 | 0.012 | 0.980 |
| 3 | rs13071962 | A/G | 0.922 | 0.014 | 1.82$\times{10}^{-9}$ | 0.015 | 0.015 | 0.330 |
| 4 | rs215412 | A/G | 1.070 | 0.012 | 3.56$\times{10}^{-9}$ | 0.017 | 0.013 | 0.180 |
| 4 | rs13107325 | T/C | 1.166 | 0.021 | 3.85$\times{10}^{-13}$ | 0.017 | 0.029 | 0.570 |
| 4 | rs7683893 | T/C | 0.941 | 0.011 | 3.40$\times{10}^{-8}$ | 0.019 | 0.013 | 0.140 |
| 5 | rs4391122 | A/G | 0.925 | 0.011 | 5.70$\times{10}^{-13}$ | -0.017 | 0.012 | 0.160 |
| 5 | rs301714 | C/G | 1.162 | 0.026 | 6.59$\times{10}^{-9}$ | 0.026 | 0.027 | 0.330 |
| 5 | rs3849046 | T/C | 1.064 | 0.011 | 1.30$\times{10}^{-8}$ | 0.008 | 0.012 | 0.510 |
| 5 | rs3112532 | A/G | 0.933 | 0.011 | 1.57$\times{10}^{-9}$ | 0.008 | 0.013 | 0.530 |
| 5 | rs11740474 | A/T | 0.939 | 0.011 | 1.50$\times{10}^{-8}$ | 0.024 | 0.012 | 0.052 |
| 6 | rs1233578 | A/G | 1.208 | 0.016 | 1.48$\times{10}^{-31}$ | -0.015 | 0.021 | 0.470 |
| 6 | rs1339227 | T/C | 0.938 | 0.011 | 2.64$\times{10}^{-8}$ | 0.010 | 0.013 | 0.410 |
| 6 | rs217289 | A/G | 0.936 | 0.011 | 1.58$\times{10}^{-9}$ | -0.003 | 0.012 | 0.800 |
| 6 | rs117074560 | T/C | 0.847 | 0.027 | 5.46$\times{10}^{-10}$ | 0.002 | 0.032 | 0.960 |
| 7 | rs12532143 | T/C | 1.084 | 0.012 | 2.33$\times{10}^{-12}$ | -0.018 | 0.013 | 0.170 |
| 7 | rs10954580 | A/G | 1.066 | 0.011 | 1.64$\times{10}^{-8}$ | -0.021 | 0.013 | 0.096 |
| 8 | rs73191547 | A/T | 0.935 | 0.012 | 4.58$\times{10}^{-9}$ | -0.023 | 0.014 | 0.094 |
| 8 | rs78895722 | C/G | 0.832 | 0.032 | 7.34$\times{10}^{-9}$ | 0.041 | 0.030 | 0.170 |
| 8 | rs35612961 | A/G | 0.926 | 0.014 | 3.37$\times{10}^{-8}$ | 0.012 | 0.018 | 0.520 |
| 8 | rs7815859 | A/C | 1.087 | 0.013 | 4.33$\times{10}^{-10}$ | -0.002 | 0.015 | 0.880 |
| 8 | rs4129585 | A/C | 1.081 | 0.011 | 7.70$\times{10}^{-13}$ | -0.017 | 0.013 | 0.170 |
| 9 | rs7033189 | A/G | 0.937 | 0.012 | 1.55$\times{10}^{-8}$ | -0.019 | 0.014 | 0.170 |
| 10 | rs7893279 | T/G | 1.121 | 0.018 | 7.33$\times{10}^{-11}$ | 0.030 | 0.019 | 0.130 |
| 10 | rs72829007 | T/G | 1.185 | 0.029 | 6.00$\times{10}^{-9}$ | 0.027 | 0.033 | 0.420 |
| 10 | rs7085104 | A/G | 1.102 | 0.011 | 1.37$\times{10}^{-17}$ | 0.017 | 0.013 | 0.170 |
| 11 | rs11027857 | A/G | 1.067 | 0.011 | 2.95$\times{10}^{-9}$ | 0.003 | 0.012 | 0.800 |
| 11 | rs7951870 | T/C | 0.912 | 0.014 | 1.58$\times{10}^{-10}$ | 0.007 | 0.016 | 0.650 |
| 11 | rs10791097 | T/G | 1.080 | 0.011 | 1.61$\times{10}^{-12}$ | 0.006 | 0.013 | 0.660 |
| 11 | rs3758927 | C/G | 1.094 | 0.014 | 4.27$\times{10}^{-11}$ | -0.001 | 0.015 | 0.930 |
| 12 | rs1024582 | A/G | 1.104 | 0.011 | 2.71$\times{10}^{-18}$ | 0.022 | 0.013 | 0.086 |
| 12 | rs672107 | A/T | 0.939 | 0.011 | 3.02$\times{10}^{-8}$ | -0.008 | 0.013 | 0.520 |
| 12 | rs61937595 | T/C | 0.866 | 0.021 | 2.37$\times{10}^{-12}$ | 0.010 | 0.024 | 0.680 |
| 12 | rs4766428 | T/C | 1.068 | 0.011 | 2.76$\times{10}^{-9}$ | 0.002 | 0.013 | 0.850 |
| 14 | rs10149407 | T/C | 1.068 | 0.012 | 2.35$\times{10}^{-08}$ | 0.017 | 0.013 | 0.190 |
| 14 | rs221903 | T/C | 1.068 | 0.011 | 5.63$\times{10}^{-9}$ | -0.010 | 0.013 | 0.450 |
| 14 | rs2332700 | C/G | 1.081 | 0.013 | 4.20$\times{10}^{-10}$ | 0.005 | 0.014 | 0.710 |
| 14 | rs35229468 | T/C | 1.089 | 0.012 | 6.62$\times{10}^{-13}$ | 0.020 | 0.013 | 0.130 |
| 15 | rs2414718 | A/G | 1.072 | 0.011 | 2.35$\times{10}^{-10}$ | -0.012 | 0.013 | 0.340 |
| 15 | rs28681284 | T/C | 0.906 | 0.014 | 2.26$\times{10}^{-13}$ | 0.002 | 0.016 | 0.920 |
| 15 | rs783540 | A/G | 0.941 | 0.011 | 2.49$\times{10}^{-8}$ | -0.005 | 0.013 | 0.700 |
| 15 | rs67119537 | T/C | 1.088 | 0.012 | 5.62$\times{10}^{-13}$ | -0.008 | 0.014 | 0.580 |
| 15 | rs4702 | A/G | 0.925 | 0.011 | 4.90$\times{10}^{-12}$ | 0.012 | 0.013 | 0.380 |
| 16 | rs1076884 | C/G | 1.080 | 0.013 | 1.55$\times{10}^{-9}$ | 0.014 | 0.015 | 0.350 |
| 16 | rs12933068 | A/G | 0.941 | 0.011 | 2.88$\times{10}^{-8}$ | 0.010 | 0.012 | 0.430 |
| 17 | rs4523957 | T/G | 1.067 | 0.011 | 1.25$\times{10}^{-8}$ | 0.003 | 0.013 | 0.820 |
| 18 | rs9636107 | A/G | 0.923 | 0.011 | 1.86$\times{10}^{-13}$ | 0.017 | 0.012 | 0.160 |
| 19 | rs72986630 | T/C | 1.156 | 0.024 | 1.47$\times{10}^{-9}$ | -0.042 | 0.033 | 0.210 |
| 19 | rs2916068 | A/G | 0.937 | 0.011 | 1.05$\times{10}^{-8}$ | -0.009 | 0.013 | 0.510 |
| 19 | rs2053079 | A/G | 0.930 | 0.013 | 1.32$\times{10}^{-8}$ | -0.005 | 0.015 | 0.730 |
| 20 | rs6065094 | A/G | 0.928 | 0.012 | 1.11$\times{10}^{-10}$ | 0.018 | 0.013 | 0.160 |
| 22 | rs5757730 | A/G | 0.930 | 0.011 | 1.56$\times{10}^{-10}$ | -0.024 | 0.012 | 0.053 |
| 22 | rs9607782 | A/T | 1.094 | 0.013 | 2.60$\times{10}^{-12}$ | 0.020 | 0.014 | 0.170 |

^a^Allele: risk/reference allele; ^b^LogOR: log odds ratio; ^c^SE: standard error

Chr: chromosome, SCZ: schizophrenia, SNP: single nucleotide polymorphism, T2D: type 2 diabetes
